# Transcriptional control of ROS homeostasis by KUODA1 regulates cell expansion during leaf development

**DOI:** 10.1038/ncomms4767

**Published:** 2014-05-07

**Authors:** Dandan Lu, Ting Wang, Staffan Persson, Bernd Mueller-Roeber, Jos H.M. Schippers

**Affiliations:** 1 Max Planck Institute of Molecular Plant Physiology, 14476 Potsdam-Golm, Germany; 2 Department of Molecular Biology, Institute of Biochemistry and Biology, University of Potsdam, 14476 Potsdam-Golm, Germany; 3 Plant Cell Biology Research Centre, School of Botany, University of Melbourne, Parkville, Victoria 3010, Australia; 4 Institute of Biology I, RWTH Aachen University, 52074 Aachen, Germany

## Abstract

The final size of an organism, or of single organs within an organism, depends on an intricate coordination of cell proliferation and cell expansion. Although organism size is of fundamental importance, the molecular and genetic mechanisms that control it remain far from understood. Here we identify a transcription factor, KUODA1 (KUA1), which specifically controls cell expansion during leaf development in *Arabidopsis thaliana*. We show that *KUA1* expression is circadian regulated and depends on an intact clock. Furthermore, KUA1 directly represses the expression of a set of genes encoding for peroxidases that control reactive oxygen species (ROS) homeostasis in the apoplast. Disruption of *KUA1* results in increased peroxidase activity and smaller leaf cells. Chemical or genetic interference with the ROS balance or peroxidase activity affects cell size in a manner consistent with the identified KUA1 function. Thus, KUA1 modulates leaf cell expansion and final organ size by controlling ROS homeostasis.

## Abstract (web summary)

During plant development, organ size is controlled by cell proliferation and expansion, but the molecular mechanisms involved are unclear. Here, Lu *et al.* show that leaf cell expansion is controlled by the KUA1 transcription factor that acts in a circadian manner and modulates the expression of genes encoding cell wall-localized peroxidases.

## Graphical abstract

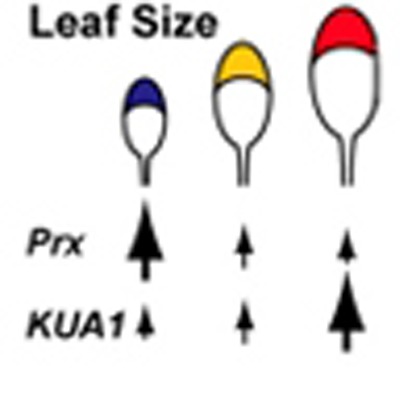

Multicellular organisms grow through a coordinated balance between cell proliferation and expansion. Alterations to this balance typically lead to abnormal development, as in the case of cancer^1^. In plants, organ size is astonishingly constant within a given species and environment. However, large differences are observed when comparing one species with another, indicating that organ size is under genetic control^2^. Plant leaves are initially established by meristematic cell proliferation and in a second phase by cell expansion without further divisions^3^. Both phases are regulated by a multitude of genetic pathways, in which a fine-tuned balance between positive and negative regulators, for example, transcription factors (TFs), plays a central role^4^. In contrast to mammalian cells, plant cells are encased by a cell wall that gives structural support. Not surprisingly, cell expansion is affected by alterations in cell wall content and architecture^5^,^6^,^7^. Such alterations may be mediated by biosynthetic and/or remodelling proteins, including expansins, a class of proteins located in plant cell walls^8^,^9^, xyloglucan endotransglucosylase/hydrolases (XETs/XTHs)^10^, and also by peroxidases (Prxs), which modulate the level of reactive oxygen species (ROS)^11^. Still, the mechanistic details of their transcriptional regulation, and therefore of plant cell expansion, remain largely unknown.

ROS orchestrate downstream signalling cascades in many different organisms, from bacteria to animals, to direct developmental processes^12^. ROS are typified as chemically reactive compounds that contain oxygen, including oxygen ions and peroxides. These compounds play important roles in the regulation of plant growth^13^. Plasma membrane-located NADPH oxidases are the most abundant ROS-producing enzymes in the expanding cell wall^14^. The activity of three members of this family has, among others, been linked to root growth^15^. For example, the root hair defective2 (RHD2) protein is required for root hair initiation and expansion^15^. Prxs represent another class of ROS-related proteins that are widely distributed between plant tissues and have functions that involve a range of different substrates^16^. In the plant apoplast, Prxs may act as hydrogen peroxide (H_2_O_2_)-consuming and/or phenol-oxidizing enzymes, which typically affect lignin formation in the secondary cell wall^17^. Although ROS can both stimulate and inhibit cell expansion^13^, the genetic regulation and/or mechanistic details are often not clear. Recently, the bHLH TF UPBEAT1 (UPB1) was found to modulate the balance between cell proliferation and differentiation by repressing *Prx* genes in *Arabidopsis thaliana* roots^18^. Inhibition of peroxidase activity by applying the chemical inhibitor salicylhydroxamic acid (SHAM) to roots or treatment with hydrogen peroxide (H_2_O_2_) resulted in reduced meristem cell number and length of the first cortical cell of the root. In contrast, *upb1-1* mutants displayed a significantly increased meristem cell number and length of the first cortical cell. This indicates that H_2_O_2_ scavenging by root peroxidases controls indeterminate root growth^18^.

In contrast to roots, leaf growth is determinate and the final size depends on a tight interplay between cell division and expansion. It would therefore be anticipated that overall leaf growth and size is controlled differently from root growth. Here we show that a MYB-like TF, KUODA1 (KUA1), modulates leaf organ size by controlling the expression of *Prxs*. We further prove causality between peroxidase activity and organ size through chemical and genetic means, thereby providing a mechanism for how final leaf organ size depends on apoplastic ROS turnover. Hence, KUA1 directly controls ROS-mediated cell expansion during leaf development to set the final size of the organ.

## Results

### KUODA1 positively regulates cell expansion

**Figure 1:**
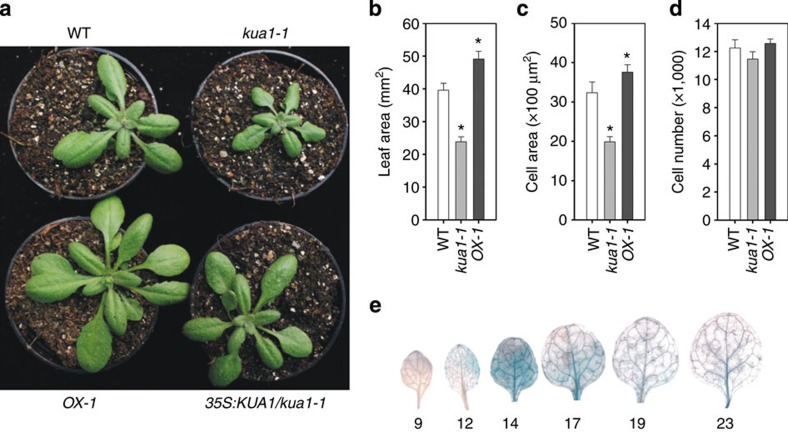
KUA1 is a positive regulator of cell expansion. (**a**) Images of 28-day-old wild type (WT), *kua1-1*, *35S:KUA1* *(OX-1)* and *35S:KUA1/kua1-1* plants, respectively. (**b**–**d**) Measurements of (**b**) leaf size, (**c**) mesophyll cell size and (**d**) cell number. Values represents means±s.d. (*n*=20). *P<0.05, Student’s *t*-test. Leaf and cell size data for a second independent overexpression line (*OX-2*) and the complementation line (*35S:KUA1/kua1-1*) is shown in Supplementary Fig. 1. (**e**) *pKUA1::GUS* expression pattern during leaf development. Activity is observed starting at the tip on day 12 and is present throughout the leaf blade at day 14 but becomes restricted towards the base at day 17, overlapping with the phase of leaf expansion.

To identify gene networks controlling organ size in *Arabidopsis*, we performed a reverse genetics screen for genes encoding TFs that are specifically induced during leaf expansion based on transcriptome data^3^. We found one T-DNA insertion mutant, which affected a *MYB*-like gene (AT5G47390) (Supplementary Fig. 1a,b), to have reduced leaf size as compared with wild type (WT; Fig. 1a,b). Complementation of the mutant with the gene’s complementary DNA (cDNA) under control of the CaMV 35S promoter verified that the observed phenotype was caused by the loss of the TF. As overexpression of the *MYB-like* gene (OX) resulted in enlarged leaves, we termed the gene *KUODA1* (*KUA1*; Chinese for ‘enlarge’ or ‘expand’). Leaf size is the sum of cell number and cell size. Most mutants known to affect leaf size act on cell proliferation, or on both cell proliferation and expansion rather than cell expansion alone^19^,^20^. To determine changes at the cellular level, mature (that is, not anymore expanding) first true leaves from soil-grown plants were harvested at 22 days after sowing (DAS). By microscopic analysis, we determined the number and area of mesophyll cells in the subepidermal layer and found that the *kua1-1* leaves had reduced cell size (65% of WT), while the number of cells was unaffected (Fig. 1c,d). Moreover, the reduction in cell size was equivalent to the reduction in leaf size of the *kua1-1* mutant. In contrast, overexpression of *KUA1* resulted in a significantly increased leaf area (*OX-1*, 123% of WT; *OX-2*, 119% of WT) and cell size (*OX-1*, 118% of WT; *OX-2*, 114% of WT), while the number of cells were unaffected (Fig. 1b–d, Supplementary Fig. 1c–e). Consistently, the smaller cell size of the *kua1-1* mutant was restored after complementation.

**Figure 2:**
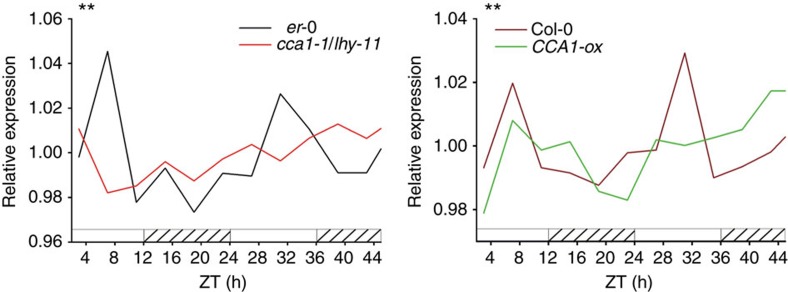
The circadian clock regulates the timing of *KUA1* expression. Expression profile of *KUA1* from 16-day-old plants entrained under 12-h light/12-h dark photocycles and transferred to constant light (LL). Sampling was performed every 4 h for three independent biological replicates. *CCA1-ox* is in the Col-0 background, and *cca1-1*/*lhy-11* is in the L*er*-0 background. Lines represent mean expression values in four genotypes: Col-0, *CCA1-ox*, L*er*-0 and *cca1-1*/*lhy-11*. One-way ANOVA (effect of time in Col-0 and L*er*-0) for *KUA1* was significant (**P<0.01). White bars represent day, and striped bars the subjective night.

Dicot leaves exhibit diel growth patterns with maximal expansion rates in the morning that are dictated by the circadian clock^21^. As *KUA1* regulates leaf growth, we anticipated that its expression might follow such rhythm. With the DIURNAL tool^22^, we found a diurnal expression pattern with maximal transcript abundance of *KUA1* in the morning (Supplementary Fig. 2a). This pattern was confirmed both at the transcript and translational level in leaves of 15-day-old plants (Supplementary Fig. 2b). Morning-phased gene expression is controlled by two MYB TFs, circadian clock associated 1 (CCA1) and late-elongated hypocotyl (LHY), which affect leaf growth rate^23^,^24^. To investigate whether *KUA1* expression depends on the clock, we performed a 48-h expression profiling on 16-day-old plants grown under 12-h light/12-h dark (LD) photocycles released to continuous light (LL) in *cca1-1*/*lhy-11*, *CCA1-ox* and WT plants^25^. Phase-specific expression of *KUA1* is lost in both *CCA1-ox* and *cca1-1*/*lhy-11* plants (one-way analysis of variance (ANOVA), *P*<0.01; Fig. 2), indicating that its diurnal transcript accumulation depends on an intact clock. The *KUA1* promoter contains a CCA1 binding site 658 bp before the transcriptional start site (Supplementary Fig. 3a), similar to the one found in the promoter of the CCA1 direct target gene *pseudo-response regulator 9* (ref. 26). Moreover, the CCA1 binding site motif was also found in upstream regulatory sequences of *KUA1* orthologues from several other species, including *Glyma17g15330* from *Glycine max* and *Os01g09280* from *Oryza sativa* (Supplementary Fig. 3a). Together with the observation that *CCA1* and *LHY* expression peaks several hours before that of *KUA1*, this suggests that *KUA1* might represent a direct clock output.

Next, we determined the spatio-temporal regulation of *KUA1* expression during leaf development. Activity of a *pKUA1::GUS* reporter construct was not observed before 12 DAS, when staining began to appear at the tip of the leaf (Fig. 1e). Of note, cell proliferation and cell enlargement proceed from the leaf apex to the base^27^. Furthermore, the transition from proliferation to expansion occurs relatively abrupt^28^, resulting in a phase of leaf development during which almost all cells undergo expansion^3^. Consistently, GUS staining intensity at 14 DAS was observed throughout the leaf blade and decreased thereafter in a basipetal manner, indicating that *KUA1* is specifically induced during leaf expansion. At maturity, weak GUS staining was observed in vascular tissue, which is in line with previously reported cell-specific expression data for *KUA1* in companion cells (Supplementary Fig. 3b).

The KUA1 protein contains a central MYB-like DNA-binding domain and at the N-terminal side a CCHC zinc-finger domain (Supplementary Fig. 1f). Furthermore, two regulatory motifs were detected, a nuclear localization sequence and a transcriptional repression domain, R/KLFGV^29^. To test whether KUA1 is targeted to the nucleus *in planta*, the *kua1-1* mutant was transformed with a KUA1-GFP fusion protein expressed from the 35S promoter. The translational fusion construct complemented the growth phenotype of the *kua1-1* mutant and resulted in a nuclear localized signal of the fusion protein (Supplementary Fig. 1g).

### KUODA1 is a transcriptional regulator of ROS-related genes

**Figure 3:**
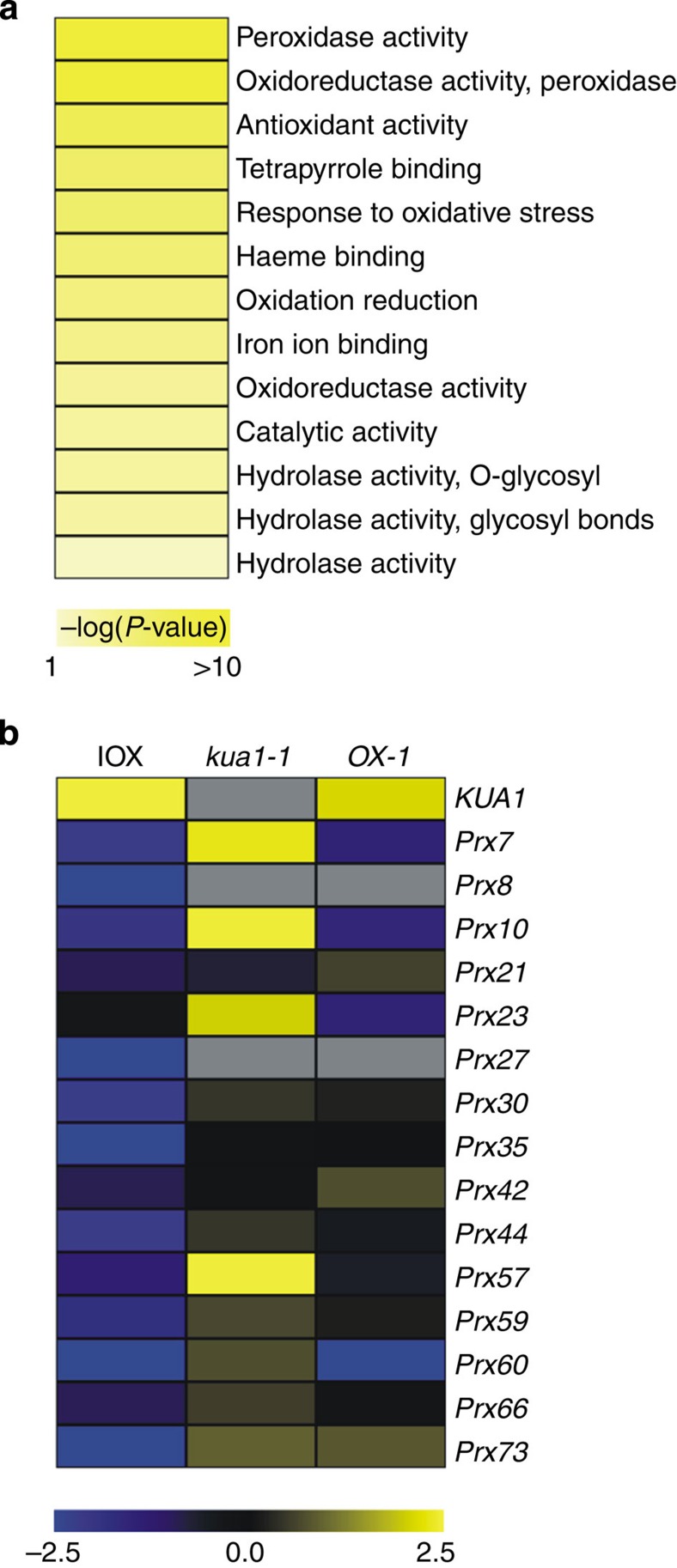
Identification of KUA1-regulated genes. (**a**) Enriched gene ontology (GO) categories among genes that are negatively regulated by KUA1. (**b**) Expression profile of *Prx* genes on alteration of *KUA1* expression levels as determined by qRT-PCR. Data represent mean ΔΔCT from three independent biological replicates (see Supplementary Table 2). Yellow indicates an increase in expression, blue indicates a decrease in expression; scale bar shows Log_2_ fold changes (FC). Naming of peroxidase genes after Valério *et al.*^36^

As *KUA1* is specifically induced during leaf expansion, we envisaged that its downstream target genes have a role in setting organ size. To identify KUA1-regulated genes, we made use of an estradiol-inducible overexpression (IOX) system^30^ and subjected *KUA1-IOX* seedlings after induction to global transcriptome analysis. After 4 h of *KUA1* induction, 198 genes were differentially expressed (twofold change, Student’s *t*-test *P*<0.05) of which 107 were upregulated and 91 were downregulated (Supplementary Data 1). As KUA1 contains a transcriptional repression domain, we mainly focused our analysis on genes downregulated by the TF. To investigate possible biological functions of these genes, we identified significantly enriched gene ontology (GO) categories among the changed genes^31^. The most significantly enriched GO category of genes negatively regulated by KUA1 was ‘peroxidase activity’ (*P*<10^−12^) (Fig. 3a, Supplementary Table 1). Other enriched categories included ‘antioxidant activity’ (*P*<10^−6^) and ‘response to oxidative stress’ (*P*<10^−6^).

For the genes positively regulated by KUA1, the most enriched GO category was ‘glutathione transferase activity’ (*P*<10^−6^) (Supplementary Fig. 4a, Supplementary Table 1), indicating that KUA1 affects ROS homeostasis. In addition, ‘cell wall’ (*P*<10^−4^) and ‘endomembrane system’ (*P*<10^−6^) GO categories were over-represented among the upregulated genes, suggesting a role of KUA1 in cell wall remodelling^5^.

We verified the expression of a select number of differentially expressed genes by qRT–PCR (Supplementary Table 2). *Glutathione* *S-transferase (GSTU)* genes were upregulated on inducible overexpression of *KUA1*, but a mild effect was observed on the expression of these genes in *OX-1* plants (Supplementary Fig. 4b). Still, *GSTU* genes were downregulated in *kua1-1* together with several TF genes, including *ZAT12*, *REVEILLE8* (*RVE8*) and a bHLH (AT1G10585). In contrast, inducible overexpression of *KUA1* resulted in the downregulation of more than 10 *Prx* genes (Fig. 3b). Consistently, constitutive overexpression of *KUA1* represses these genes, while in the *kua1-1* mutant several showed enhanced expression, most prominently *Prx7*, *Prx10*, *Prx23* and *Prx57*. As KUA1 appears to act as a transcriptional repressor, we used a transient luciferase reporter gene assay to validate this. To this end, the promoter of *Prx7* was cloned directly upstream of the firefly luciferase open reading frame and transformed into *Arabidopsis* protoplasts (Supplementary Fig. 5a). Co-transformation of the reporter construct with a *35S:KUA1* construct resulted in a significant downregulation of the luciferase signal, indicating that KUA1 *in vivo* acts as a transcriptional repressor of *Prx7*. Fusion of a GAL4 activation domain with the KUA1 protein overcomes the repressive effect of KUA1 and causes a significant induction of the *Prx7* reporter. Taken together, KUA1 acts as a transcriptional repressor and modulates the expression of ROS-related genes.

### KUODA1 is a direct regulator of peroxidase genes

**Figure 4:**
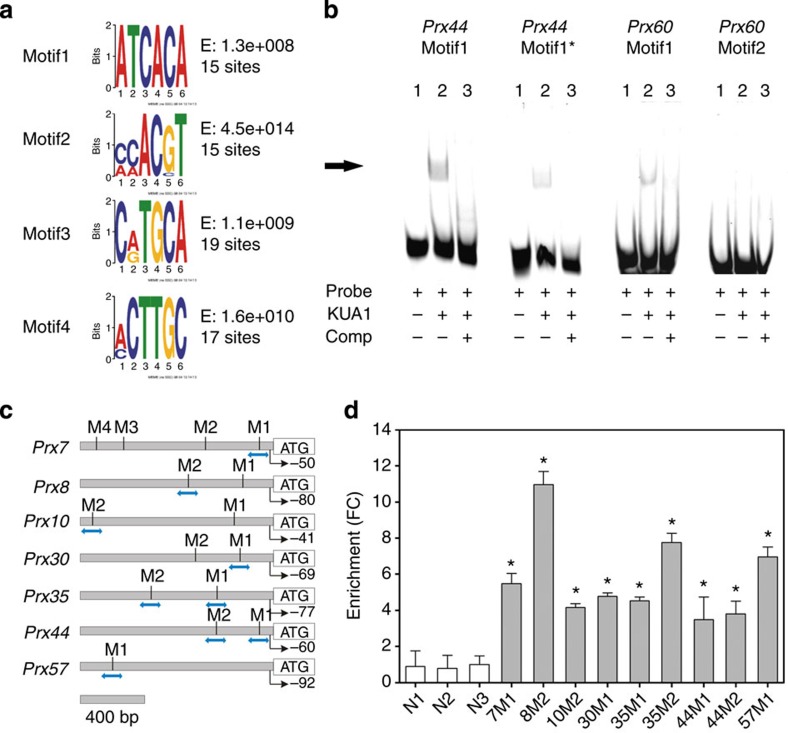
KUA1 is a direct negative regulator of peroxidase gene expression. (**a**) Identified 6-mer motifs (Motif1-4) enriched in upstream promoter sequences of the differentially expressed *Prx* genes in *KUA1-IOX* microarray experiment, as detected by the MEME Suite and TAIR motif finder. Indicated are the E-value, representing the statistical significance of the motif, and the number of sites contributing to the construction of the motif^32^. (**b**) EMSA performed with probes for Motif1 and Motif2 (A). Incubation of KUA1 protein with oligonucleotides spanning Motif1 from the promoters of *Prx44* or *Prx60* causes a band shift (arrow), which disappears on competition (‘comp’) with unlabelled probe (100-fold molar excess). A mutation in Motif1 from *Prx44* (Prx44 Motif1*) from ATCACA to GTCACA reduced binding affinity. No retention was observed with probes based on Motif2 from *Prx60*. (**c**) KUA1-binding sites (M; as based on Motif1) in upstream promoter regions of selected *Prx* genes. Blue arrows indicate amplified regions in ChIP-qPCR experiments. Positions of the transcriptional start sites are indicated by arrows with turning lines. (**d**) ChIP-qPCR results from *35S:KUA1-GFP*/*kua1-1* plants for seven *Prx* promoters and three negative controls. M1 and M2 represent KUA1 binding sites as shown in **c**; *Prx* genes are indicated by numbers. N1, N2 and N3 represent genomic control regions lacking KUA1 binding sites. Values represent average enrichment (FC) of three independent biological replicates. The amounts of immunoprecipitated genomic DNA were normalized to the input fraction. The fold enrichments for immunoprecipitation of the KUA1-GFP DNA complex by an anti-GFP antibody were calculated over control (IgG precipitated) samples for each analysed region. *P<0.05, Student’s *t*-test. Error bars indicate s.e. (*n*=3).

The upstream promoter sequences of the differentially expressed *Prxs* were screened for over-represented 6-mer motifs to identify a potential DNA-binding site for KUA1 using the MEME Suite^32^ and TAIR motif finder ( www.arabidopsis.org). This analysis resulted in the identification of several abundant motifs (Fig. 4a). These include ATCACA (Motif1), which represents an unknown motif, and AAACGT (Motif2), which is identical to the SORLREP2 motif that is present in the promoters of light-regulated genes^33^. Furthermore, an element similar to the RY/G-box was found, C(A/G)TGCA (Motif3), which renders seed-specific expression^34^, and the (A/C)CTTGC (Motif4), which is similar to the iron-deficiency response element^35^. We used an electrophoretic mobility shift assay (EMSA) to assess whether KUA1 binds to any of these motifs. We found that KUA1 can bind a DNA probe spanning the unknown ATCACA motif (Motif1) in the promoters of both *Prx44* and *Prx60*, while no binding was found with probes containing the AAACGT motif (Motif2) from *Prx60* (Fig. 4b). In addition, a probe with a point mutation in Motif1 from *Prx44* causing the exchange of the first base into a G (GTCACA; Motif1*) was less strongly retained in the gel as compared with probes with the original motif. Subsequently, we tested whether *Prxs* are direct KUA1 targets by performing a chromatin immunoprecipitation (ChIP) assay with the functional GFP-tagged KUA1 protein (*35S:KUA1-GFP*/*kua1-1*). To this end, primers spanning genomic regions containing the identified *cis*-element were used (Fig. 4c). We found that KUA1 associated with the promoters of *Prx7*, *Prx8*, *Prx10*, *Prx30*, *Prx35*, *Prx44* and *Prx57* (Fig. 4d). In addition, no enrichment was detected for three negative controls (promoter segments of three ROS-related genes whose expression levels were unaffected by KUA1) or in the further upstream regions in the promoter of *Prx30* (Supplementary Fig. 5b). Thus, KUA1 directly interacts with seven *Prx* loci causing repression of their expression.

### Peroxidase activity is linked to cell size

**Figure 5:**
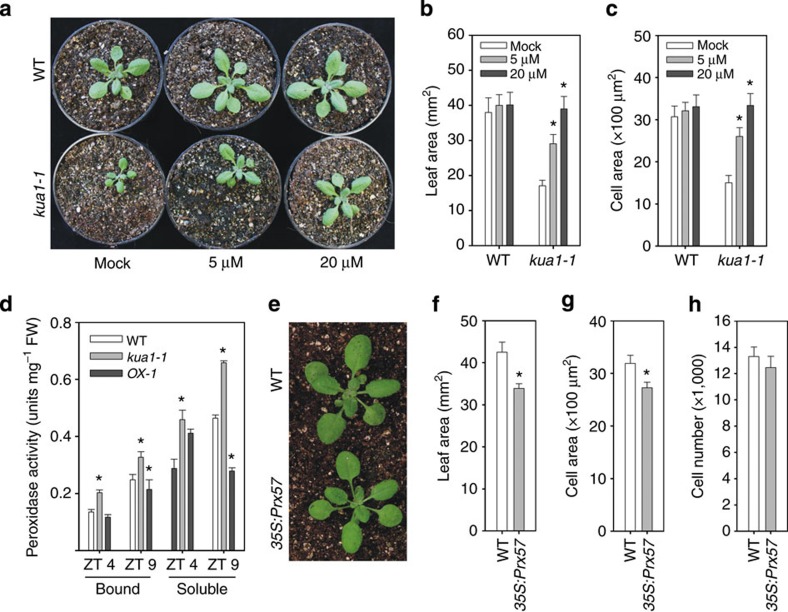
Peroxidase activity is a major determinant of cell size. (**a**) Images of 22-day-old wild-type (WT) and *kua1-1* plants treated at day 14 with SHAM. (**b**–**c**) Measurements of (**b**) leaf size and (**c**) cell size. Values represents means±s.d. (*n*=20). *P<0.05, Student’s *t*-test. (**d**) Peroxidase activities of ionically bound and soluble protein fractions were determined for wild type (WT), *kua1-1* and *35S:KUA1* at ZT4 and ZT9. Values represent means±s.d. (*n*=4). *P<0.05, Student’s *t-*test. FW, fresh weight. (**e**) Images of 22-day-old wild-type (WT) and *35S:Prx57* plants. (**f**–**h**) Measurements of (**f**) leaf size, (**g**) mesophyll cell size and (**h**) cell number. Values represent means±s.d. (*n*=20). *P<0.05, Student’s *t*-test.

Prxs can stimulate as well as restrict plant growth^11^. The enhanced expression of *Prxs* in *kua1-1* and their repression by KUA1 suggest that increased peroxidase activity might be symptomatic for the observed phenotype. To test this, we treated 14-day-old plants with SHAM, a peroxidase inhibitor, and measured the size of the first leaf pair at 22 DAS. SHAM treatment of the *kua1-1* mutant resulted in an increased plant size in a concentration-dependent manner (Fig. 5a). Similar, but less prominent, effects were observed for WT. A single application of 20 μM SHAM fully restored expansion growth of the first leaf pair in *kua1-1*, while 5 μM showed a partial recovery as compared with WT (Fig. 5b,c). Since Class III peroxidases have been implicated in the production of H_2_O_2_ in the apoplast^11^,^36^, we stained plants with the 3,3′-diaminobenzidine and found an increased intensity in the *kua1-1* mutant as compared with WT (Supplementary Fig. 6a). Quantitative determination of the H_2_O_2_ concentration revealed a significant increase in *kua1-1* leaves, while significant lower levels were detected in *OX-1* plants, as compared with wild type (Supplementary Fig. 6b). In contrast, no difference in the levels of superoxide was found between the different lines (Supplementary Fig. 6c). Moreover, treatment of *kua1-1* with the H_2_O_2_ scavenger KI reverted the mutant phenotype (Supplementary Fig. 6d–g). These results indicate that increased peroxidase activity and apoplastic H_2_O_2_ production are causative for the reduced growth.

As leaf growth is under control of the diel cycle, we measured cell wall peroxidase activity at Zeitgeber times ZT4 and ZT9. *KUA1* had the highest expression level at ZT4 (Supplementary Fig. 2). Consistent with a negative regulation of *Prx* expression, we found lower peroxidase activity in the WT at ZT4 as compared with ZT9 for both the ionically bound and soluble fraction (Fig. 5d). Furthermore, *kua1-1* showed significantly higher peroxidase activity at both time points, while overexpressing plants showed significantly lower activity at ZT9. To further confirm that Prxs regulate leaf growth, we obtained an overexpression line for *Prx57* (ref. 18), which showed increased peroxidase activity (Supplementary Fig. 6h). Interestingly, leaf and cell size in plants with increased Prx57 activity was significantly reduced, while cell number was not affected (Fig. 5e–h). These results imply that KUA1 modulates cell size through the action of cell wall-located Prxs.

Prxs are mainly known to control extensibility of the cell wall by causing crosslinking or cleaving of cell wall polysaccharides^11^,^36^. Still, it is possible that the changes in *Prx* expression cause other cell wall alterations. We therefore determined the cell wall compositions in the different genotypes, including the *35S:Prx57* line. Neither cellulose, the major component of the cell wall, was affected by altering the expression level of *KUA1* (Table 1), nor was that of uronic acids. In dicots, the hemicellulose network mainly consists of xyloglucan^37^, which is known to be enzymatically loosened by the action of expansins, endoglucanases and endotransglycosylases to allow for turgor-driven growth^38^,^39^. Among the neutral sugars measured, we only found an increase in the level of glucose for the *KUA1* overexpressing line (Table 1). These data indicate that changes in *KUA1* expression have only limited effect on the composition of the cell wall.

## Discussion

**Figure 6:**
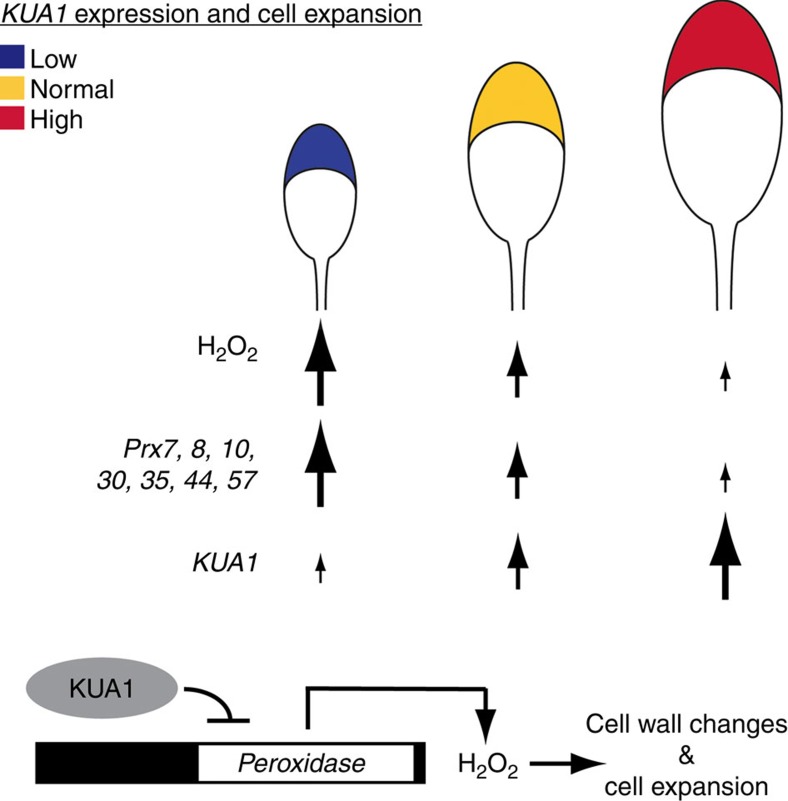
Proposed model of KUA1-dependent regulation of leaf cell size. In this model, the expression level of *KUA1* is positively correlated with whole-leaf and leaf cell size, but negatively with peroxidase activity. A high expression of *KUA1* (red) results in the repression of seven peroxidase *Prx* genes, which allows for cell expansion due to lower H_2_O_2_ levels and subsequently reduced crosslinking of the cell wall. In contrast, restriction of leaf size can be achieved by downregulation of *KUA1* (blue), which results in an increased peroxidase activity and a stiffening of the cell wall, inhibiting cell expansion.

Plant tissue growth depends on cell proliferation and directed cell expansion. Although several gene regulatory networks that control leaf primordia initiation and proliferation have been uncovered^4^, those controlling cell expansion remain largely elusive. Here, we identified *kua1-1*, which has smaller leaves due to a decrease in cell size but not cell number. In contrast, *KUA1* overexpression resulted in increased cell size and larger leaves, while the number of cells was unaffected. Thus, KUA1 is a specific regulator of leaf cell size in plants (Fig. 6). Through expression profiling on inducible overexpression lines, we found that KUA1 negatively regulates the expression of *Prx* genes. Furthermore, by ChIP and EMSA assays, we demonstrated that KUA1 directly regulates the expression of seven *Prx* genes. This repression alters the apoplastic ROS balance, establishing the means to physically control cell expansion in leaves (Fig. 6).

Apoplastic Prxs are known to either restrict or promote cell expansion^11^,^17^,^18^. These apparent contradictory effects are linked to the regulatory modes under which the Prxs are working. On one hand, the Prxs may favour elongation by generation of oxygen radicals or restrict growth by the generation of H_2_O_2_. In the first scenario, cell wall polymers may be cleaved by the radicals and thus promote cell wall loosening and therefore growth. In contrast, an increase in H_2_O_2_ typically leads to crosslinking phenolics and promotes rigid crosslinks between extensins making the cell wall stiffer^11^. KUA1 effectively represses *Prx* expression and activity in leaves, which promotes cell expansion. While this promotion is clearly linked to changed levels of apoplastic H_2_O_2_, it is important to note that changes in H_2_O_2_ also can affect the pool of oxygen radicals^40^. Notably, at least in the case of KUA1, the inhibitory effect of Prx, mediated via H_2_O_2_, appears to be the causal link for regulation of leaf cell expansion.

Organ size is typically underpinned by a balance between cell proliferation and growth^3^. However, this balance is largely depending on tissue and cell type. This may readily be seen in plant organ growth, where leaf growth is typically restricted to a set number of expanding cells, whereas root growth is a result of continuous cell proliferation and cell expansion^18^,^28^. Hence, the determinate and indeterminate growth of leaves and roots, respectively, should be regulated by different molecular and genetic means. Interestingly, Prxs were recently shown to affect *Arabidopsis* root growth^18^. Here, a superoxide/H_2_O_2_ equilibrium regulates the size of proliferation and differentiation zones. An enhanced expression of *Prx* genes in roots lacking the TF UPB1 was associated with an increased number of meristematic cells and an increase in length of the first mature cortical cells^18^. Thus, enhanced Prx activity promotes cellular proliferation and differentiation in roots. In our study, we show that repression of *Prx* expression by KUA1 enhanced leaf cell expansion, but did not cause an increase in leaf cell proliferation. Furthermore, as both SHAM and H_2_O_2_ treatments reduce the size of the first cortical root cell^18^, the root-localized Prxs are presumably needed to maintain low H_2_O_2_ levels to promote root cell expansion. In contrast, here we show that inhibition of leaf peroxidase activity increases leaf size, suggesting that in leaves the apoplastic Prxs mainly produce H_2_O_2_, which potentially results in cell wall crosslinking^11^. Hence, the impact of Prxs, or more precisely their effect on the H_2_O_2_ level, appears to have largely opposing effects on organ growth in plants.

Leaf expansion rates in dicots follow a diel rhythm^21^, and this correlates with the accumulation of *KUA1* transcript during the morning. Moreover, *KUA1* transcript cycling depends on a functional clock, as in *cca1-1/lhy-11* and *CCA1-ox* plants the time-of-day-specific expression of *KUA1* is lost. Interestingly, the promoter of *KUA1* and its orthologues contain a CCA1 binding site (Supplementary Fig. 3a), indicating that the expansion growth controlled by KUA1 could be directly regulated by the clock. Previously, we demonstrated that ROS homeostasis and ROS-responsive gene expression are under the control of CCA1 (ref. 25). Here we demonstrate that KUA1 controls apoplastic ROS homeostasis to mediate cell expansion. Of note, leaf expansion rates in dicots are the highest in the morning^24^, which correlates with the increased expression level of *KUA1* during this time of the day. Thus, to obtain high expansion rates during the morning, *KUA1* is induced to repress *Prx* gene expression. In addition, the promoter of *KUA1* contains several abiotic stress-related *cis*-elements (Supplementary Fig. 3a). As expression of *KUA1* is affected by various abiotic stresses (Supplementary Fig. 3c) it is likely that it functions to modulate growth in a changing environment.

In conclusion, we have identified a mechanism in which KUA1 regulates Prx activity to control final leaf cell size. Despite that *Prxs* are commonly required for the modulation of H_2_O_2_ levels in the apoplastic space, there are remarkable differences in the molecular implementation of their action in the regulation of leaf and root growth.

## Methods

### Plant material and growth conditions

*Arabidopsis thaliana* Columbia-0 (Col-0) was used as a wild type. The T-DNA insertion line *kua1-1* (GK-783B02) was obtained from the Nottingham *Arabidopsis* Stock Centre. Plant growth and β-estradiol treatments were done as previously described^41^. In short, *Arabidopsis* plants were grown in soil (Einheitserde GS90; Gebrüder Patzer, Sinntal-Jossa, Germany) in a growth chamber with a 16-h day (120 μmol m^−2^ s^−1^) and a day/night temperature of 22/16 °C and 60/75% relative humidity. In tissue culture, seedlings were grown in half-strength Murashige and Skoog medium (0.5 MS) supplemented with 1% sucrose and solidified with 0.7% agar under a 16-h day (140 μmol m^−2^ s^−1^)/8-h night regime (22 °C). SHAM and KI treatments were done by applying a foliar spray to 14-day-old soil-grown plants. GUS staining was performed according to Jefferson^42^.

### Construction of plasmids

To generate overexpression constructs and C-terminal GFP fusions of *KUA1*, the coding sequence (CDS) was amplified from leaf cDNA by PCR and cloned into pENTR/D (Invitrogen) with the primers listed in Supplementary Table 3. Sequence-confirmed ENTRY clones were used for recombination with pK7FWG2 vector to construct a fusion with the GFP coding region at the C-terminal part of the TF ( http://gateway.psb.ugent.be)^43^. The clones containing the stop codon were used for generating a CaMV 35S construct by recombination with vector pK7WG2 (ref. 43).

The promoter region directly upstream of the start codon of *KUA1* (1,050 bp) was amplified from genomic DNA and cloned into pENTR/D and verified by sequencing. Thereafter, the clone was recombined with the GATEWAY compatible vector pKGWFS7 that contains a fused reporter gene consisting of eGFP and the *E. coli* β-glucuronidase (GUS)^43^. End clones were selected by restriction analysis and confirmed by sequencing before plant transformation.

For the establishment of an inducible overexpression vector the *KUA1* CDS was amplified by PCR with primers that contain an *Xho*I or *Spe*I site (Supplementary Table 3). PCR products were ligated overnight by TA cloning into pGEM-T Easy vector (Promega). After sequence confirmation, the vector was digested and the *Xho*I-*Spe*I fragment containing the CDS was ligated into the pER8 vector^30^.

### Plant transformation and selection of transgenic plants

Constructs were transformed into *Arabidopsis* Col-0 wild type or the *kua1-1* mutant using *Agrobacterium tumefaciens* (strain GV3101)-mediated transformation employing a modified floral dip method^44^. T_0_ seeds were selected on MS medium containing the appropriate antibiotic. Subsequently, stable transgenic T_3_ lines were established based on a segregation analysis. Transgenic lines showing either an increased expression level by qPCR or reporter gene signal were used for detailed analysis in this study.

### Microscopic analysis

Cleared first true leaves of 22-day-old plants were used to measure leaf area, cell size and cell number according to Nguyen *et al.*^41^

### Total and polysomal RNA isolation

Total RNA was extracted from leaves or seedlings using the RNeasy kit (Qiagen). Before cDNA synthesis with RevertAid First-strand cDNA Synthesis kit (Fermentas), the isolated RNA was treated with DNase I (Ambion). Quantitative real-time PCR was done using Power SYBR Green PCR Master Mix (Applied Biosystems). *ACTIN2* (AT3G18780) was used as reference gene (Supplementary Table 3). Oligonucleotides for qPCR were designed with the webtool QUANTPRIME^45^. Relative transcript abundance was determined by the comparative C_T_ method^46^. Polysomes from *35S::HF-RPL18* plants (15 DAS) were immunoprecipitated using powdered tissue and extracted in polysome extraction buffer as previously described^47^,^48^.

### Gene expression analysis

For microarray experiments total RNA was isolated from 4-h mock or 15 μM estradiol-treated *KUA1-IOX* plants. Two independent biological replicates for each treatment were used for hybridization to *Arabidopsis* whole-genome Affymetrix ATH1 GeneChip (ATLAS Biolabs). Expression data were submitted to the NCBI Gene Expression Omnibus (GEO) repository ( www.ncbi.nlm.nih.gov/geo/) under accession number GSE50520.

### ChIP assay

*35S:KUA1-GFP*/*kua1-1* plants were grown for 12 days on MS media. Whole-shoot tissue was fixed with formaldehyde and for the ChIP assay the EpiQuick kit (Epigentek Group Inc, NY, USA) was used as described in Lai *et al.*^25^

### Motif analysis and EMSA

To identify conserved promoter motifs among the differentially expressed *Prx* genes, 1,000-bp upstream sequences were extracted using Phytozome^49^. Promoter analysis was performed using the MEME program with the following settings: one or more occurrences per sequence, a 6-base motif length, and 15 as the minimum number of sites to be detected in all promoters tested^32^. Next to that, the AGI codes of the *Prx* genes were used as a query set for TAIR motif analysis ( www.arabidopsis.org). Motifs reported by both tools were compared with known *cis*-regulatory elements using the webtool ATCOECIS^50^. Subsequently, several motifs were tested empirically by using an EMSA as previously described^51^.

In short, the CDS of KUA1 was cloned into the pF3A WG Flexi vector (Promega), which contains translation enhancer sequences from the barley yellow dwarf virus (BYDV), an RNA plant virus, that facilitates *in vitro* expression of proteins in wheat germ extracts. For the *in vitro* expression, 5.0 μg of plasmid DNA was added to TNT SP6 High Yield Wheat Germ Mastermix (Promega) containing 1 μl FluoroTec Green Lys (Promega). After 2 h of incubation at 30 °C, synthesized protein was analysed by SDS-PAGE and detection was performed with a Typhoon Scanner (GE Healthcare).

For gel-shift assays, Cy5-labelled probes were generated based on 35–40 bp long regions derived from the *Prx* promoters (Supplementary Table 3). DNA probes were established by annealing forward and reverse oligonucleotides in equal-molar ratios and slowly cooling them down from 95 to 30 °C. Probes were diluted to a final concentration of 500 fmol and kept in amber tubes until use. Binding reactions were performed using the LightShift Chemiluminescent Assay Kit (Pierce, Rockford, USA). The incubated protein-DNA mixture was separated on a 5% native polyacrylamide gel after which the Cy5 signal was captured using a Typhoon Scanner.

### Peroxidase activity assay and ROS quantification

Proteins were extracted from leaves according to Bindschedler *et al.*^52^ Peroxidase activity was determined spectrophotometrically at 405 nm using 2,2′-azino-bis(3-ethylbenzothiazoline-6-sulphonic acid) diammonium salt as a substrate. Determination of H_2_O_2_ was done by using the Amplex Red Hydrogen Peroxide/Peroxidase Assay Kit (Invitrogen) as described previously^25^. NBT staining and measurement of superoxide levels were done according to Tsukagoshi *et al.*^18^

### Cell wall biochemical analysis

For cell wall analysis, the first leaf pair of soil-grown plants at 15 DAS was extracted, and crude cell wall material of six independent biological replicates was used for neutral sugar analysis, uronic acid and cellulose measurements as described^53^.

### Transactivation assay

A 1-kb promoter fragment of *Prx7* was amplified and cloned into the p2GWL7.0 vector^43^. Furthermore, a *35S:KUA1* and *35S:GAD:KUA1* fusion constructs (the latter containing the GAL4 activation domain) were created as described before^54^. Subsequently, protoplast transformation and measurement of luciferase signal were done as reported previously^41^.

## Author contributions

J.H.M.S. and B.M.-R. designed the experiments. D.L. and J.H.M.S. performed most experiments and data analyses, T.W. and S.P. performed cell wall analysis, D.L., B.M.-R. and J.H.M.S. wrote the manuscript; J.H.M.S. and S.P. revised the manuscript.

## Additional information

**Accession codes**: The microarray expression data have been deposited in the NCBI Gene Expression Omnibus (GEO) database under accession code GSE50520.

**How to cite this article:** Lu, D. *et al.* Transcriptional control of ROS homeostasis by KUODA1 regulates cell expansion during leaf development. *Nat. Commun.* 5:3767 doi: 10.1038/ncomms4767 (2013).

## Supplementary Material

Supplementary Figures, Tables and Reference Supplementary Figures 1-6, Supplementary Tables 1-3 and Supplementary Reference.

Supplementary Data 1 Indentification of KUA1 regulon. Genes differentially expressed upon induction of KUA1. Results are based on two biological replicates.

## Figures and Tables

**Table 1:** Cell wall composition is mildly affected by *KUA1* expression level.

	**WT**	***kua1-1***	***OX-1***	***35S:Prx57***
Cel	98.44±14.5	109.48±14.7	105.20±3.3	96.14±3.7
UAs	45.14±7.8	49.86±4.4	48.04±3.4	43.91±6.3
Rha	6.82±1.0	5.80±1.6	5.70±1.4	5.79±1.7
Fuc	1.15±0.1	0.93±0.2	1.05±0.2	1.02±0.3
Ara	7.89±1.0	6.88±1.2	7.34±1.5	7.94±2.0
Xyl	7.35±0.8	5.89±1.3	6.28±1.6	6.13±1.8
Man	2.55±0.4	1.83±0.5	2.18±1.0	1.97±0.5
Gal	16.51±1.9	16.88±3.0	16.62±2.8	15.70±3.4
Glc	8.46±1.3	11.40±4.1	**13.98±2.8**	8.56±2.2

Ara, arabinose; Cel, cellulose; Fuc, fucose; Gal, galactose; Glc, glucose; Man, mannose; Rha, rhamnose; UAs, uronic acids; Xyl, xylose.

First leaves were collected at 18 DAS and extracted. Values are displayed as μg/mg dry weight±s.d. *N*=6 biological reps, each with three technical reps. Bold values indicate *P<0.01, two-tailed *t*-test.
