# Supplementary material for: Transcriptional control of ROS homeostasis by KUODA1 regulates cell expansion during leaf development
Source: Nat Commun. 2014 May 7;5:3767. doi: 10.1038/ncomms4767 (PMC4024751; doi:10.1038/ncomms4767)
Supplement: Supplementary Figures, Tables and Reference — Supplementary Figures 1-6, Supplementary Tables 1-3 and Supplementary Reference. [file ncomms4767-s1.pdf]

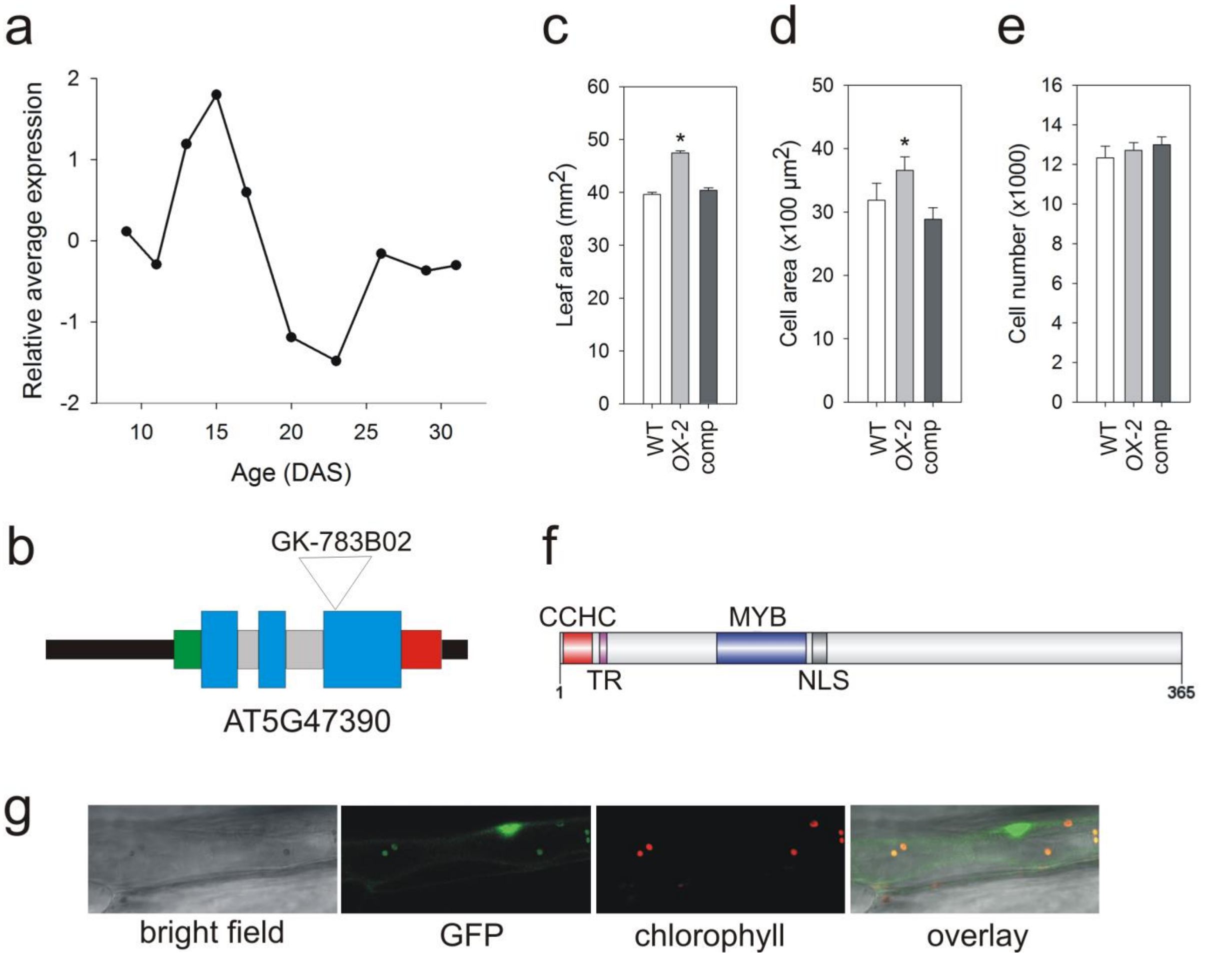

Supplementary Figure 1 | Identification of KUA1 and its role during leaf growth.

(**a**) Expression profile of *KUA1* during the development of the first leaf pair. Data were extracted from Beemster et al. (2005). Values represent the average relative expression level during leaf development. Days 12 to 17 correspond to the expansion phase. DAS, days after sowing. (**b**) Figure indicating the position of the T-DNA insertion site in the *kua1-1* mutant. Blue boxes: exons; green: 5' UTR; grey: introns; red: 3' UTR. (**c**-**e**) Measurements of (**c**) leaf area, (**d**) mesophyll cell size, and (**e**) cell number of wild type, a second independent *35S:KUA1* line (*OX-2*) and the complementation (comp) line. Data were determined on first-pair leaves at 22 DAS. Values represent means ± SD (n = 20). **P* < 0.05, Student's *t* test. (**f**) Scheme representing the KUA1 protein containing a CCHC zinc finger domain, a transcriptional repression (TR) domain (Ikeda et al., 2009), the MYB domain (MYB), and a nuclear localization signal (NLS). The KUA1 protein has 365 amino acids. (**g**) Subcellular localization of KUA1 in plants. The *kua1-1* mutant was transformed with a *35S:KUA1-GFP* construct. Green fluorescence indicates GFP, red signal indicates auto-fluorescence of chloroplasts.

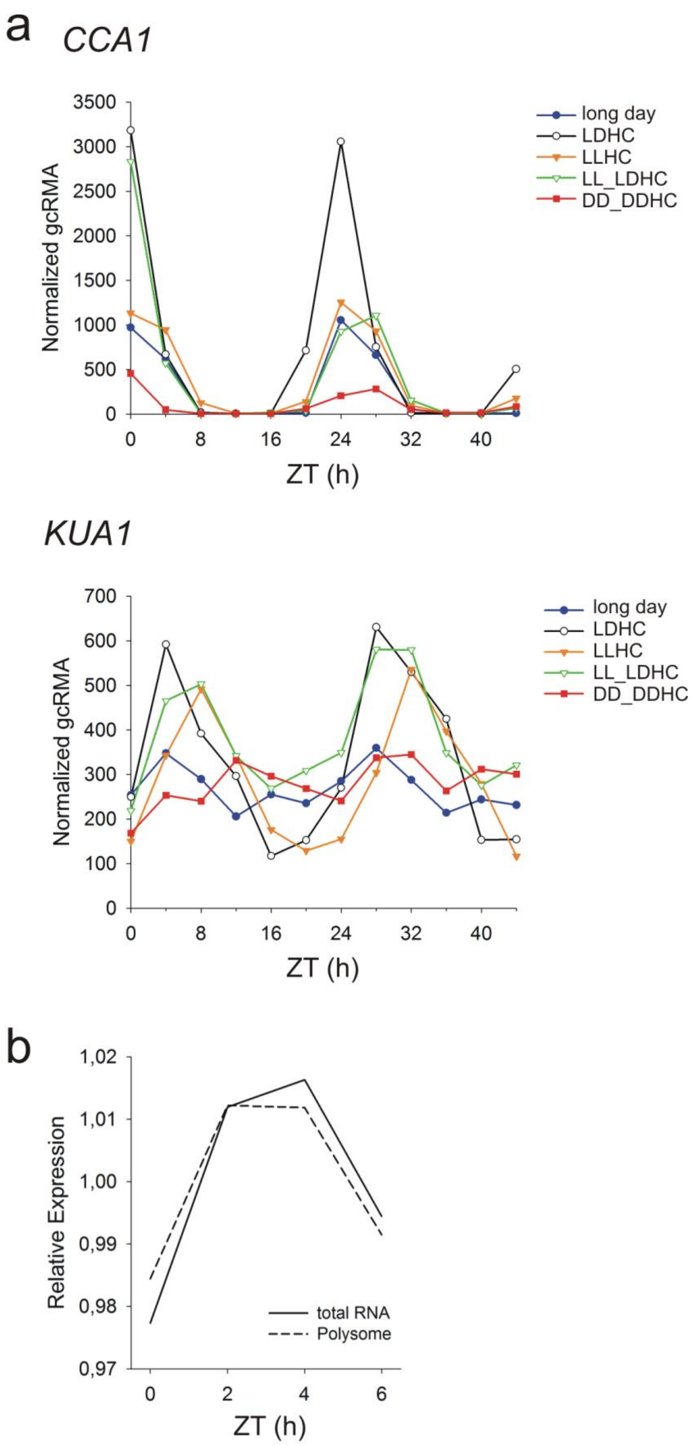

Supplementary Figure 2 | Diurnal regulation of *KUA1* expression.

(**a**) Expression profiles of *CCA1* and *KUA1* grown in diurnal (long day = 16 h : 8 h, light : dark; LDHC = 12 h : 12 h, light : dark; LLHC = constant light) or circadian conditions (LL_LDHC = release into constant light after growth in LDHC; DD_DDHC = release into constant darkness after constant darkness). gcRMA refers to Genechip Robust Multiarray Averaging of values. Data were extracted from webtool DIURNAL (Mockler et al., 2007). ZT

0 represents start of the day (onset of light). (**b**) Morning expression profile of *KUA1* obtained from 15-day-old soil-grown Col-0 plants under a 16-h light/8-h dark photocycle. Expression profiling was performed both on total RNA as well as on the polysome-bound mRNA fractions. For each time point, data represent the means of three biological replicates.

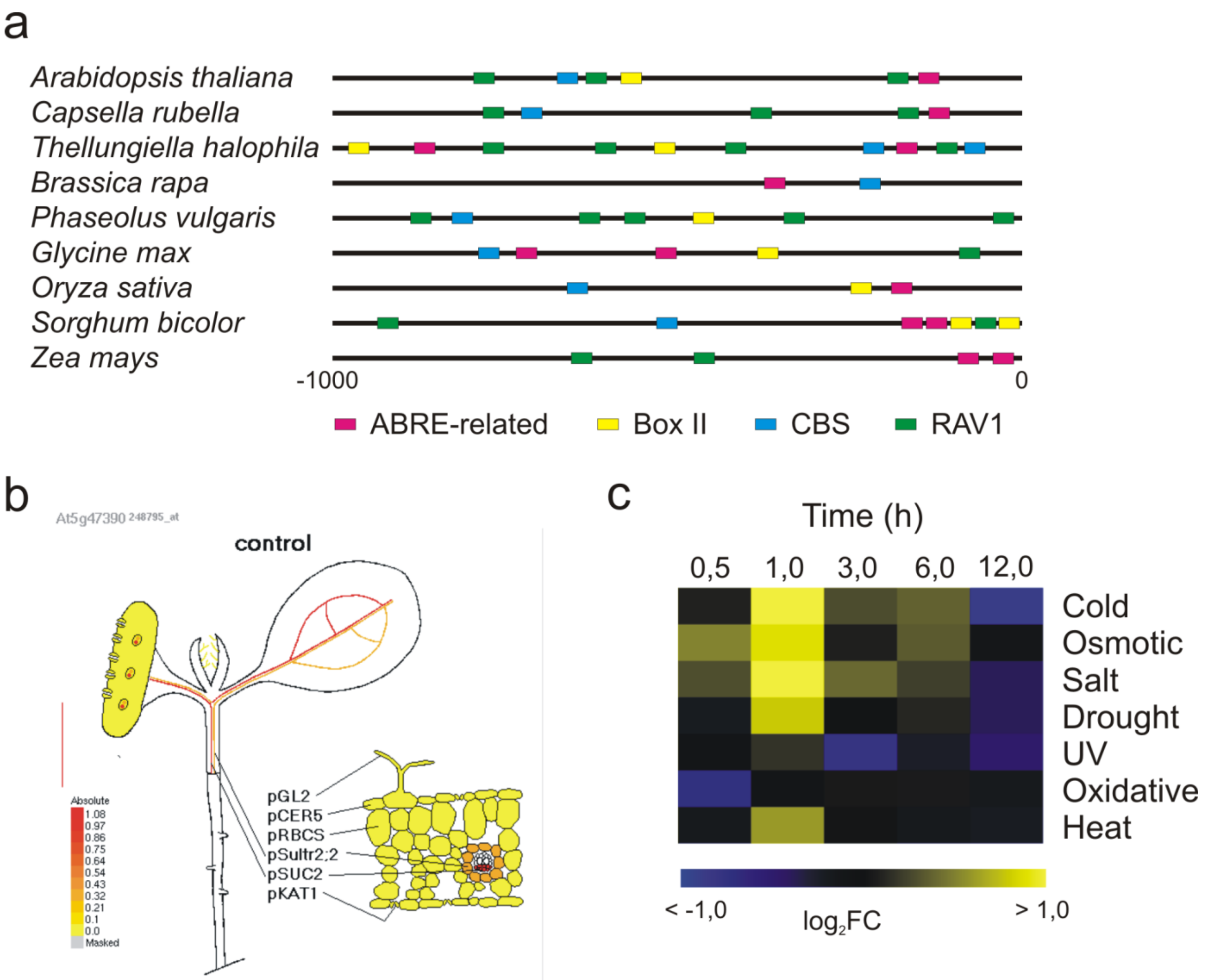

Supplementary Figure 3 | *KUA1* promoter analysis and expression response to abiotic stress.

(**a**) Shown are *cis*-elements that are present in the promoter of *KUA1* and its orhologs from *Capsella rubella* (Carubv10026634m), *Thellungiella halophila* (Thhalv10000932m), *Brassica rapa* (Bra022127), *Phaseolus vulgaris* (Phvul.003G222900), *Glycine max* (Glyma17g15330), *Oryza sativa* (Os01g09280), *Sorghum bicolor* (Sb01g029020) and *Zea mays* (GRMZM2G034110)**.** Shown are the first 1000 bp upstream of the transcription start site for each gene. Boxes indicate the position of the respective *cis*-elements, including the ABRE-motif (pink), Box II (yellow), CBS motif (blue) and the RAV1 binding site (green). (**b**) Relative mRNA abundance of *KUA1* in cell-type specific leaf translatomes of Arabidopsis as visualized by the eFP platform (efp.ucr.edu\; Mustroph et al., 2009). Notably, *KUA1* mRNA is most abundant in companion cells (pSUC2). (**c**) *KUA1* expression is modulated during different types of abiotic stresses. Heatmap represent the log_2FC change in expression and is based on microarray data from Killian et al. (2007). Experiments were performed on 18 day old plants with cold treatment at 4°C, 300 mM mannitol treatment for osmotic stress and 150 mM NaCl for salt stress, exposure to drought or UV-light for 15 minutes, oxidative stress was induced by exposure to 10 µM methyl viologen, heat treatment was performed at 38°C.

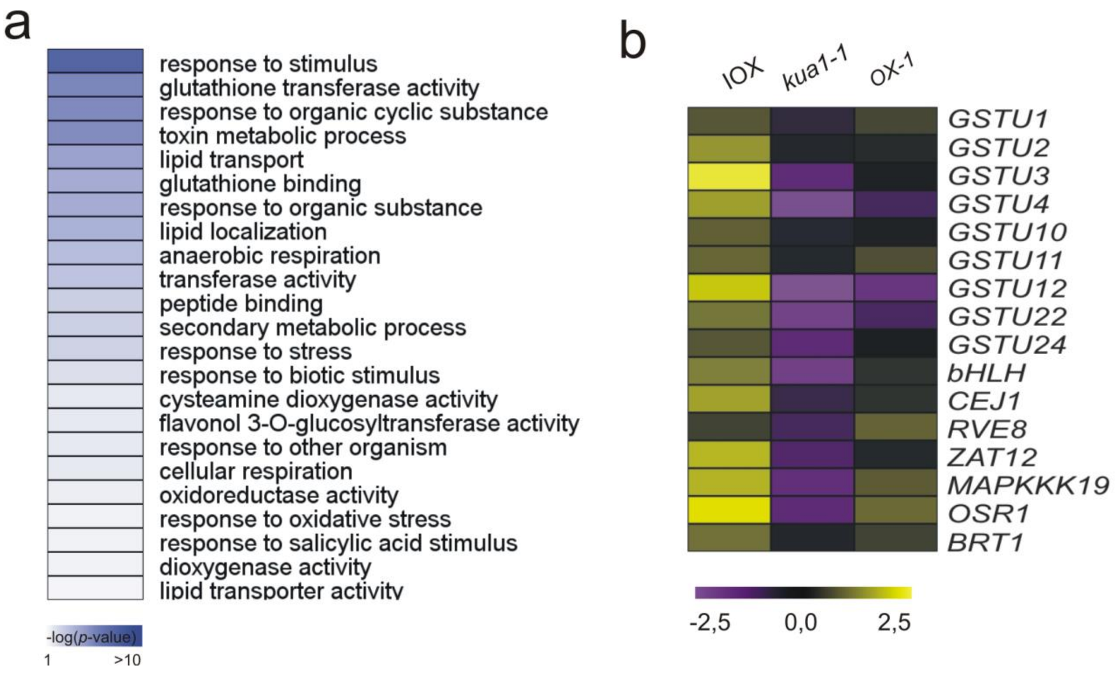

Supplementary Figure 4 | Genes upregulated by KUA1.

(**a**) Enriched Gene Ontology (GO) categories among genes that are positively regulated by KUA1. (**b**) Expression profile of KUA1 upregulated genes as determined by qRT-PCR. Yellow indicates an increase in expression, purple indicates a decrease in expression; scale bar shows Log_2 fold changes (FC). For the *KUA1-IOX* line (IOX), 15-day-old seedlings were treated for 4 h with estradiol and compared to mock-treated seedlings. For *kua1-1* and the overexpression line *OX-1,* leaves from 15-day-old soil grown plants were used for expression profiling and compared to WT.

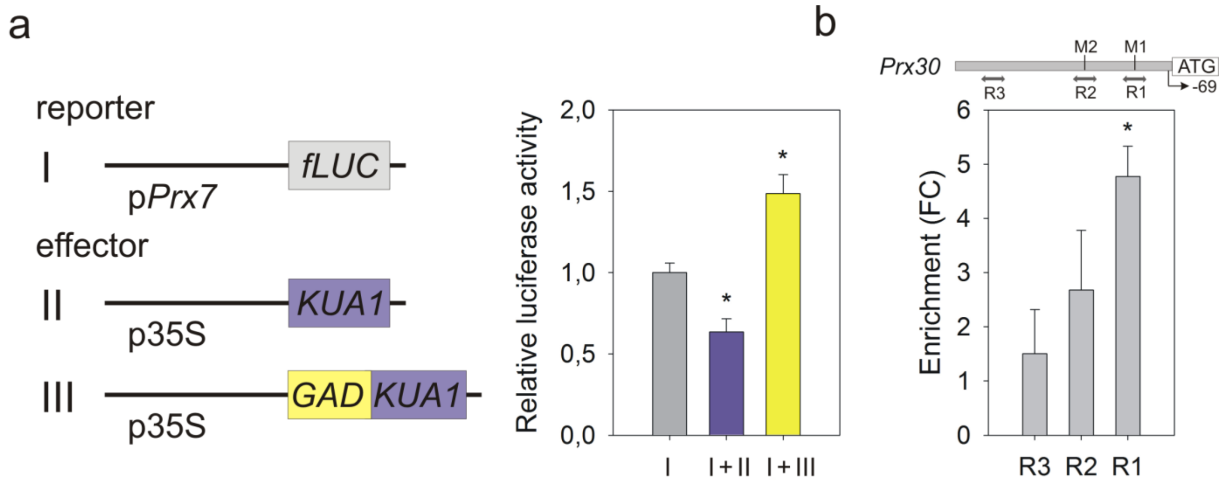

Supplementary Figure 5 | KUA1 acts as a transcriptional repressor.

(a) Transactivation assay to test the ability of KUA1 to repress gene expression via interaction with the promoter of *Prx7* in Arabidopsis protoplasts. On the left a schematic representation of the reporter and effector constructs is given. (I) The promoter of *Prx7* was cloned in front of the firefly luciferase gene as reporter, (II) effector construct containing the KUA1 CDS under the control of the CaMV 35S promoter, and (III) an effector that contains the KUA1 CDS in fusion with the yeast GAL4 activation domain (GAD). On the right the relative luciferase signal detected after transformation with the respective constructs is shown. Co-transformation of the *Prx7* reporter with KUA1 resulted in a repression of the luciferase signal while incubation with the GAD:KUA1 fusion resulted in a significant activation of the reporter. n = 4. An asterisk (*) indicates a significant difference ($P \leq 0.05$, Student´s *t* test) to the luciferase signal obtained with the reporter construct only. **(b)** ChIP promoter scanning results for *Prx30*. A schematic overview is given for the first 1200 bp upstream of the transcription start site of *Prx30* and the three tested regions are indicated as R1 to R3. Of note, R1 corresponds to the region shown in Fig. 4d. Values represent average enrichment (FC) of three independent biological replicates. The amounts of immunoprecipitated genomic DNA were normalized to the input fraction. The fold enrichments for immunoprecipitation of the KUA1-GFP DNA complex by anti-GFP antibody were calculated over control (IgG precipitated) samples for each analyzed region. *$P < 0.05$, Student's *t* test. Error bars indicate SE.

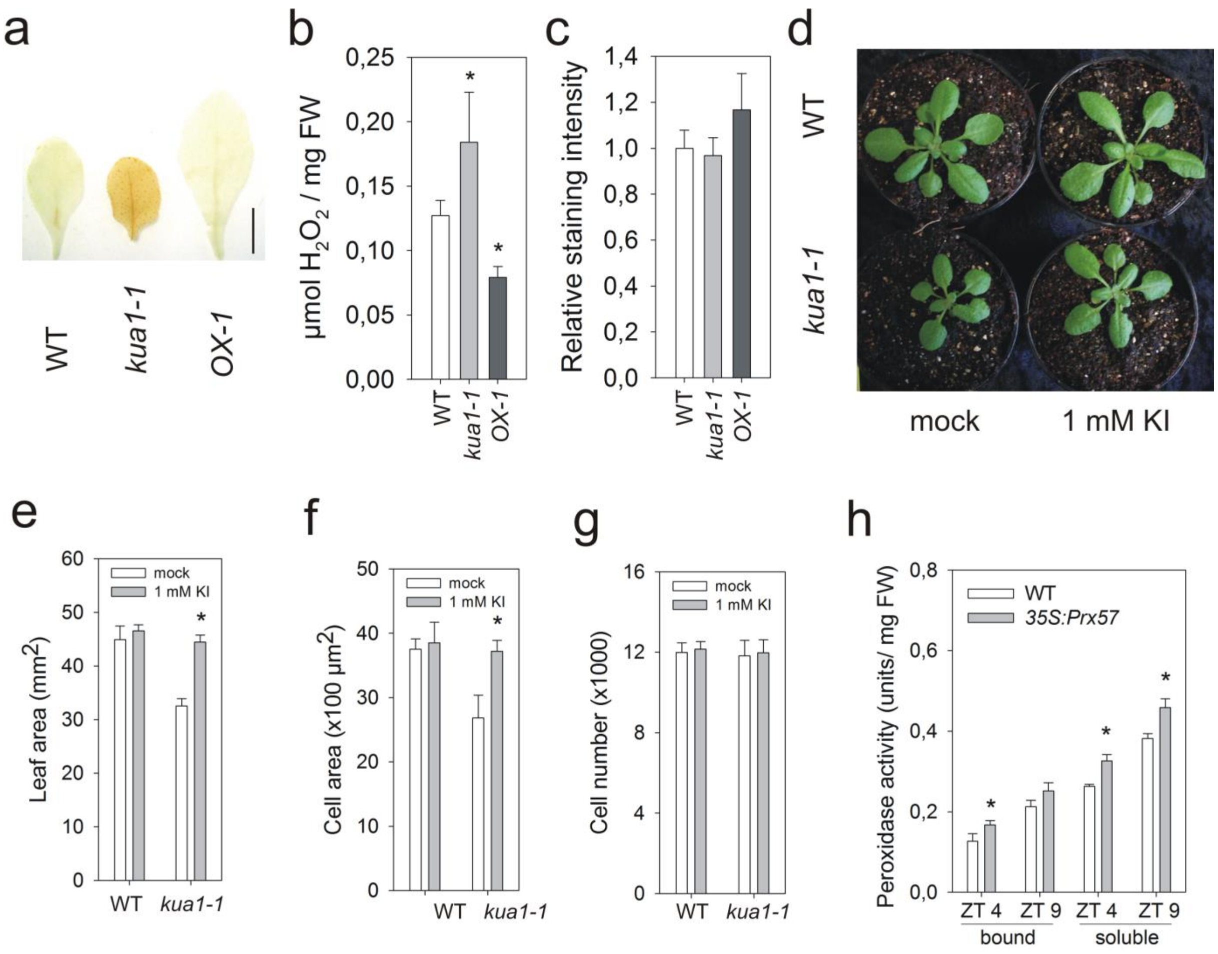

Supplementary Figure 6 | *kua1-1* mutants have increased H_2O_2 levels and scavenging reverts the reduced-growth phenotype.

(**a**) DAB stain for hydrogen peroxide in wild type (WT), *kua1-1* and the overexpression line *OX-1*. Shown are cleared leaves of 28-day-old soil-grown plants that were collected in the morning. (**b**) H_2O_2 levels in wild type, *kua1-1* and *35S:KUA1*. Values represent mean concentrations of three independent biological replicates. Asterisks indicate significant difference (Student's *t* test; $^*P < 0.05$). (**c**) Levels of superoxide are shown as relative staining intensity for nitroblue tetrazolium (NBT) in leaves of WT, *kua1-1* and *OX-1*. (n = 20). Data were normalized to the mean staining intensity obtained for WT leaves. (**d**) Images of 22-day-old WT and *kua1-1* plants treated at day 14 with a foliar spray of 1 mM KI solution. Similar results were obtained with 0.5 mM KI. (**e**-**g**) Measurements of (**e**) leaf area, (**f**) mesophyll cell size and (**g**) cell number after KI treatment. Data were determined on first-pair leaves from 22-day-old plants. Values represent means ± SD (n = 20). $^*P < 0.05$, Student's *t* test. (**h**) Peroxidase activity of the ionically bound and soluble protein fractions from cell wall extracts were determined for wild-type (WT) and *35S:Prx57* plants at ZT4 and ZT9. Proteins were extracted from leaves of 15-day-old soil grown plants. Values represent means ± SD (n = 3). $^*P < 0.05$, Student's *t* test.

Supplementary Table 1 | GO Enrichment Analysis of Genes Differentially Expressed upon the Induction of *KUA1*

GO term	Enrichment	*p*-value	Subset ratio	Description
**DOWNREGULATED GENES**				
Molecular function				
GO:0004601	4,98	8,77E-12	13 %	peroxidase activity
GO:0016684	4,98	8,77E-12	13 %	oxidoreductase activity, acting on peroxide as acceptor
GO:0016209	3,70	1,30E-7	13 %	antioxidant activity
GO:0046906	2,97	1,58E-6	15 %	tetrapyrrole binding
GO:0020037	2,95	7,22E-6	14 %	heme binding
GO:0005506	2,61	8,75E-5	14 %	iron ion binding
GO:0016491	1,85	4,56E-4	20 %	oxidoreductase activity
GO:0003824	0,78	0,0010	55 %	catalytic activity
GO:0004553	2,74	0,0012	10 %	hydrolase activity, hydrolyzing O-glycosyl compounds
GO:0016798	2,68	0,0017	10 %	hydrolase activity, acting on glycosyl bonds
GO:0016787	1,13	0,04	24 %	hydrolase activity
Biological process				
GO:0006979	3,23	1,75E-6	14 %	response to oxidative stress
GO:0055114	2,24	2,58E-5	20 %	oxidation reduction
Cellular compartment				
GO:0012505	1,34	7,39E-6	38 %	endomembrane system
GO:0005618	2,28	3,85E-4	14 %	cell wall
GO:0030312	2,23	5,56E-4	14 %	external encapsulating structure
GO:0005576	2,29	0,01	10 %	extracellular region
GO:0009535	2,69	0,01	8 %	chloroplast thylakoid membrane
GO:0031984	2,49	0,02	8 %	organelle subcompartment
GO:0044434	1,62	0,03	14 %	chloroplast part
GO:0010598	5,99	0,03	2 %	NAD(P)H dehydrogenase complex (plastoquinone)
GO:0044435	1,54	0,04	14 %	plastid part
**UPREGULATED GENES**				
Molecular function				
GO:0004364	5,11	3,48E-6	6 %	glutathione transferase activity
GO:0043295	5,87	1,01E-4	4 %	glutathione binding
GO:0016765	3,76	9,33E-4	6%	transferase activity
GO:0042277	4,72	0,0027	4 %	peptide binding
GO:0047800	6,79	0,02	2 %	cysteamine dioxygenase activity
GO:0047893	6,79	0,02	2 %	flavonol 3-O-glucosyltransferase activity
GO:0016702	4,84	0,03	3 %	oxidoreductase activity
GO:0051213	4,61	0,04	3 %	dioxygenase activity
GO:0005319	4,53	0,05	3 %	lipid transporter activity
Biological process				
GO:0042221	1,67	1,33E-8	37 %	response to chemical stimulus
GO:0050896	1,32	4,51E-8	47 %	response to stimulus
GO:0014070	3,86	4,98E-6	9 %	response to organic cyclic substance
GO:0009404	5,17	6,28E-6	6 %	toxin metabolic process
GO:0009407	5,17	6,28E-6	6 %	toxin catabolic process

GO:0006869	3,81	4,27E-5	8 %	lipid transport
GO:0010876	3,65	1,01E-4	8 %	lipid localization
GO:0010033	1,66	1,02E-4	24%	response to organic substance
GO:0009061	5,59	5,36E-4	4 %	anaerobic respiration
GO:0019748	2,38	0,003	11 %	secondary metabolic process
GO:0006950	1,25	0,0033	27 %	response to stress
GO:0009607	1,83	0,01	15 %	response to biotic stimulus
GO:0051707	1,76	0,02	14 %	response to other organism
GO:0045333	3,64	0,02	5 %	cellular respiration
GO:0006979	2,44	0,04	8 %	response to oxidative stress
GO:0009751	3,00	0,04	6 %	response to salicylic acid stimulus
Cellular compartment				
GO:0005886	1,10	0,01	23 %	plasma membrane

Supplementary Table 2 | Gene Expression Confirmation by qRT-PCR

Gene	*IOX*	*kua1-1*	*OX-1*
KUA1	**3.78**	n.d.	**2.11**
DOWNREGULATED GENES			
Prx7	**-2.19**	**2.31**	**-1.51**
Prx8	**-6.29**	n.d.	n.d.
Prx10	**-1.98**	**2.45**	**-1.58**
Prx21	-0.95	-0.74	0.57
Prx23	0.06	**2.02**	**-1.51**
Prx27	**-2.67**	n.d.	n.d.
Prx30	**-2.22**	0.43	0.22
Prx35	**-3.75**	-0.08	-0.01
Prx42	-0.90	-0.06	0.71
Prx44	**-2.19**	0.44	-0.47
Prx57	**-1.35**	**3.40**	-0.77
Prx59	**-1.83**	0.64	0.19
Prx60	**-3.79**	0.72	**-3.25**
Prx66	-0.95	0.53	-0.18
Prx73	**-2.85**	0.91	0.80
UPREGULATED GENES			
GSTU1	0.75	-0.68	0.56
GSTU2	**1.45**	-0.43	0.22
GSTU3	**4.49**	**-1.46**	-0.14
GSTU4	**1.56**	**-2.36**	-0.94
GSTU10	0.86	-0.51	0.07
GSTU11	0.93	-0.45	0.67
GSTU12	**1.95**	**-2.85**	**-1.71**
GSTU22	**1.11**	**-2.08**	-0.99
GSTU24	0.75	**-1.43**	0.00
bHLH	**1.22**	**-2.01**	0.33
CEJ1	**1.59**	-0.78	0.31
RVE8	0.52	-0.96	0.90
ZAT12	**1.79**	**-1.15**	0.18
MAPKKK19	**1.75**	**-1.53**	0.81
OSR1	**2.24**	**-1.42**	0.98
BRT1	**1.04**	-0.37	0.53

Data shown are mean ddC_T (Log2FC). Bold numbers indicate >2-fold changes that are significant ($P < 0.05$, Student´s t test).

Supplementary Table 3 | Primers Used in This Study

Purpose	Name	Sequence (5'-3')
Genotype	G783B02-LP	TGAGGTTTGTTCCTTCTTCATTCA
	G783B02-RP	CAAGGCTGAGCTTAGACATACCAA
	GABI-BP	ATATTGACCATCATACTCATTGC
KUA1 cDNA cloning	35S:KUA1-F	CACCATGACTCGTCGATGTTCTCA
	35S:KUA1-R	TTATAAAGCGTGTATCACGCTT
	35S:KUA1-R_NS	TAAAGCGTGTATCACGCTT
KUA1 promoter cloning	pKUA1-F	CACCGTCGCCTTACTATATGACCA
	pKUA1-R	ACCCGACCCGATTTGCTTCTCTTA
KUA1 inducible overexpression	IOX-F	CTACTCGAGGTATGACTCGTCGATGTTCTCA
	IOX-R	GTCACTAGTGCTTATAAAGCGTGTATCACGCTT
EMSA probes	Prx44M1_F	GGCTTAGAATTTCATCACAAAACTTGCAAGTA
	Prx44M1_R	TACTTGCAAGTTTTGTGATGAAATTCTAAGCC
	Prx44M1*_F	GGCTTAGAATTTCGTCACAAAACTTGCAAGTA
	Prx44M1*_R	TACTTGCAAGTTTTGTGACGAAATTCTAAGCC
	Prx60M1_F	GACATTAGAAAATCATAATGTGATCGCATGAGTGAGC
	Prx60M1_R	GCTCACTCATGCGATCACATTATGATTTTCTAATGTC
	Prx60M2_F	AGAATTGAAGAAAACATTAGAAAAACGTATCTTAT
	Prx60M2_R	ATAAGATACGTTTTTCTAATGTTTTCTTCAATTCT
qRT-PCR	AT5G47390_F	TCGCTGGTGATGGTTACGCTTC
KUA1	AT5G47390_R	TCCTCTGTCCATGGAGTTCCTTTC
	AT1G30870_F	CGGGATGTGATGCGTCTGTTCTTC
Prx7	AT1G30870_R	TAGCCGGAGATCTTCTCTCTGTCC
	AT1G34510_F	CCGAACGGTTACTGCAGCATTG
Prx8	AT1G34510_R	AGAGGGAAGCATCACAACCTTTG
	AT1G49570_F	AGAGAAGCTGTCGTCCTCACTG
Prx10	AT1G49570_R	TCGCCGTTAATGAGTCCCTTCG
	AT2G37130_F	GCGAGAGACGGTATTGTCATGTTG
Prx21	AT2G37130_R	CATCTCCCAAGTAGCTCCCTCTAC
	AT2G38390_F	TACTTCGGGACGTGCCCATTTG
Prx23	AT2G38390_R	CGCTCTGGCTAATACAGCTCTTCC
	AT3G01190_F	AAATGTGTGACCACCGGAGAGG
Prx27	AT3G01190_R	GGTGATGGCAAGTTGACTTCGTTG
	AT3G21770_F	ACTGCTAGAGACGCAGTTGTCG
Prx30	AT3G21770_R	TCCTACCGTCTCTTCTACCCGTTG
	AT3G49960_F	GATTGCTCTTTCAGCGGCTCAC
Prx34	AT3G49960_R	AGTTGGGTCCACGGAGTTGATG
	AT4G21960_F	CAAGAACACCGCTTTCTCTTGGC
Prx42	AT4G21960_R	AAAGCGACGCATCACATGACTC
	AT4G26010_F	AATGCAAGCGTGAGAGGCTACG
Prx44	AT4G26010_R	ATGCAGCCTCGAGCTGTCTCTTAG
	AT5G17820_F	TTTCCACGACTGTTTCGTTAAGGG
Prx57	AT5G17820_R	ATTCCCTGACGCTTCCGTTTGG
	AT5G19890_F	TCGCCAAATTTGTAGCCGTAAACC
Prx59	AT5G19890_R	TGTCCAAAGGTGTGAGCTCCTG
	AT5G22410_F	CAAAGATGACCGTCGCGGAAAC
Prx60	AT5G22410_R	CACTCCAATCGTATGTCCACCAAG
	AT5G51890_F	CTTGTCCTCGTACCGTGTCTTG
Prx66	AT5G51890_R	TCCAATAAGGACCACCGGACAG
	AT5G67400_F	AGAGGACATGATCGCTCTTTCAGC
Prx73	AT5G67400_R	TGTTGAACACTTTGCCACAATGGG
	AT2G29490_F	TCGATCAGACGTGGAAGAACAGTC
GSTU1	AT2G29490_R	AAATCGAGCCATGGCCTTCTC
	AT2G29480_F	ATCCCTACGAGAAGGCCATGGTTC
GSTU2	AT2G29480_R	AAGCCAACTGGTAGGATCTGCTC
	AT2G29470_F	CGACCAAACATGGACAAACAATCC
GSTU3	AT2G29470_R	CCTGAGCCCTATCATTGTGACTTG
	AT2G29460_F	GATGAACAGGTTGGACCAGTAGC

GSTU4	AT2G29460_R	TGAGCCTCCTTGATTGCAACCTC
	AT1G74590_F	ACCAACAGGTGTTTGAGGTCATGG
GSTU10	AT1G74590_R	ACAGACTTTGCTTGAGCTTCACC
	AT1G69930_F	ACCGGCGTCAAGTTCATACATCC
GSTU11	AT1G69930_R	ACCGCTTCATGGGCGTAGAATC
	AT1G69920_F	GCTCACTTCGTTGACGGAAAGTTG
GSTU12	AT1G69920_R	AGTGCCGCTAGATTCTCCATCAG
	AT1G78340_F	TTGATTTCGTGGACACCAAGCTG
GSTU22	AT1G78340_R	TTGGCTGTCTCTTGTTCCTCTCC
	AT1G17170_F	TGCCAAATTCTGGGCCGACTTC
GSTU24	AT1G17170_R	TCACCTTTGACCGCCCAAATCC
	AT1G10585_F	GGATCGAAGGATGCGCATGAAAC
bHLH	AT1G10585_R	AGGTGAGGCACTGGTAACTTGC
	AT3G50260_F	GGTGGTGCAGAACAACAAACGC
CEJ1	AT3G50260_R	AATAACCCGCCGCCAAAGTCAC
	AT3G09600_F	ACTCTTCGTGGAGCAGAAGCTG
RVE8	AT3G09600_R	TGAAGCACTGGAGGCTGTTTAGC
	AT5G67080_F	ACGGCGTTAAATTACGGTGTGC
MAPKKK19	AT5G67080_R	AGATTCCGGCGCCATGTACAAC
	AT2G41230_F	ATGCAGTAGAAGCCTTCATGTCAC
OSR1	AT2G41230_R	CCCAACGACATGTTTCCTCCATC
	AT3G21560_F	ACGATGGAAGCTGTGTCTTCCG
BRT1	AT3G21560_R	TGTAAACGGCGTCCGTGACTTG
	AT3G18780_F	TCCCTCAGCACATTCCAGCAGAT
ACT2	AT3G18780_R	AACGATTCCTGGACCTGCCTCATC
ChIP assay	Prx7M1_F	ATGCAGGCAAGTCACTCGAATA
	Prx7M1_R	TGGGTTTGGAAACTAATGTGATG
	Prx8M2_F	AAAATCTCCACACTACCATGCAC
	Prx8M2_R	AAGTGGAGTGAAAAACTGCAGAC
	Prx10M2_F	CATTTTCCATCATTTGGTTGTG
	Prx10M2_R	TACTCCGATCCCTCTCGATTTA
	Prx30M1_F	GCTGTTTTTGGCTTCTCATTTTA
	Prx30M1_R	GCCGAACAAAATGTTGTGTTTTT
	Prx35M1_F	GGATTCCACTTTCATGTTTCGAC
	Prx35M1_R	AAGTAAATGTGGATTGCCAACAA
	Prx35M2_F	TATTAAGCATTGTGTGCGGAGTT
	Prx35M2_R	TGGGTTAGATTGTGATTGTTGGT
	Prx44M1_F	ATTTACGGGGGCTTAGAATTTCA
	Prx44M1_R	CCTTCCCTCTCCTGTGAATGATA
	Prx44M2_F	GGACGGCTGAAAAGAGGTATATG
	Prx44M2_R	CACGTCTATGTCGATAGGTGGAT
	Prx57M1_F	TCTTGTTCAAATGTTTTCGCTTT
	Prx57M1_R	TCTTGTTCAAATGTTTTCGCTTT
N1	AT4G23100_F	GGATTTGATTCCACCTACTAAGAA
	AT4G23100_R	TGGTTCTGATGAACGAAAATTG
N2	AT4G26850_F	TTTGTTTTGGTCCTTTGCCTA
	AT4G26850_R	TCTTAGGTCCTTTCTTGTGGATTT
N3	AT5G59720_F	TTTCTTAAATTCCACTTCCGACCT
	AT5G59720_R	CTCAGTGAAGCGGATCTTCTTTG

Naming of peroxidase genes after Valério et al. (2004)[1].

Supplementary Reference

1. Valério, L., De Meyer, M., Penel, C. & Dunand, C. Expression analysis of the Arabidopsis peroxidase multigenic family. *Phytochemistry* **65,** 1331-1342 (2004).
